# Soft-Community Kernel Rényi Spectrum for Semantic Uncertainty Estimation in Large Language Models

**DOI:** 10.3390/e28040442

**Published:** 2026-04-14

**Authors:** Zongkai Li, Junliang Du

**Affiliations:** 1 Centre for Advanced Robotics, School of Engineering and Materials Science, Queen Mary University of London, London E1 4NS, UK; ex24128@qmul.ac.uk; 2 MoE Key Lab of Artificial Intelligence, AI Institute, Shanghai Jiao Tong University, Shanghai 200240, China

**Keywords:** semantic uncertainty estimation, LLM, Rényi entropy, kernel spectral methods, hallucination detection

## Abstract

Uncertainty estimation is critical for deploying large language models (LLMs) in safety-sensitive and decision-critical applications. Recent approaches estimate semantic uncertainty by clustering multiple sampled responses into equivalence classes and measuring their diversity via entropy-based criteria. However, existing methods typically rely on greedy hard clustering and von Neumann entropy, which suffer from sensitivity to clustering order, noise in semantic equivalence judgments, and limited control over spectral contributions. In this work, we propose a principled information-theoretic framework for LLM semantic uncertainty estimation based on soft semantic communities and kernel Rényi entropy. Given multiple generations for a query, we construct a weighted semantic graph using pairwise semantic similarity scores and infer soft community assignments via weighted graph community detection. These soft assignments induce a positive semi-definite semantic kernel that captures the distribution of semantic modes without enforcing hard equivalence relations. Uncertainty is then quantified by the Rényi entropy of the kernel spectrum, yielding a tunable measure that interpolates between sensitivity to dominant semantic modes and long-tail semantic diversity. Compared to prior von Neumann entropy-based estimators, the proposed Rényi spectral uncertainty offers improved robustness to semantic noise, reduced dependence on clustering heuristics, and greater flexibility through its order parameter. Extensive experiments on question answering tasks demonstrate that our method provides more stable and discriminative uncertainty estimates, particularly under limited sampling budgets and noisy semantic judgments.

## 1. Introduction

Large language models (LLMs) have demonstrated remarkable performance across a wide range of natural language generation tasks, including question answering, reasoning, and code generation [1,2]. Despite these advances, reliably estimating the uncertainty of LLM outputs remains a fundamental challenge, particularly in safety-critical and decision-sensitive applications such as medical consultation, scientific assistance, and automated programming [3,4].

The practical importance of semantic uncertainty estimation extends across a range of real-world LLM deployments. In medical question answering, a model may produce multiple fluent responses that differ subtly in diagnosis, treatment suggestion, or risk interpretation [5]; in legal or financial assistance, semantically distinct answers may imply different obligations, recommendations, or decisions [6]; and in retrieval-augmented generation, a model may generate responses that sound confident despite deviating from the retrieved evidence [7]. In such cases, token-level confidence alone is often insufficient, because linguistic fluency does not guarantee semantic consistency or factual reliability. This makes semantic uncertainty estimation a practically important component for trustworthy LLM deployment, especially in high-stakes settings where undetected semantic variation may lead to harmful downstream consequences.

A common approach to uncertainty estimation in LLMs is based on token-level predictive entropy, which quantifies uncertainty from the model’s output distribution over tokens [8,9]. However, token-level measures often fail to reflect uncertainty at the semantic level: multiple generated responses may differ substantially in surface form while conveying the same meaning, or conversely, appear linguistically similar while implying contradictory semantic interpretations. As a result, token entropy can severely underestimate or misrepresent the true uncertainty of a model’s answers.

To address this limitation, recent work has shifted attention toward semantic uncertainty estimation, where uncertainty is defined over the space of meanings expressed by multiple sampled generations rather than over individual tokens [10,11,12]. A representative line of work samples multiple responses for a given query, clusters them into semantic equivalence classes using natural language inference (NLI) [13,14], and computes uncertainty as the entropy of the resulting semantic distribution [5,11]. This paradigm marks an important conceptual step toward meaning-aware uncertainty estimation.

Nevertheless, existing semantic uncertainty estimators typically rely on hard, greedy clustering procedures and von Neumann entropy [15] applied to kernelized semantic representations. Such designs introduce several limitations. First, greedy hard clustering is inherently sensitive to sampling order and noise in pairwise semantic judgments, and it enforces strict equivalence relations that are often violated in practice due to ambiguity and paraphrasing variability. Second, von Neumann entropy corresponds to a Shannon entropy over the kernel spectrum and provides limited flexibility in controlling the contribution of dominant versus long-tail semantic modes. Finally, the tight coupling between clustering heuristics and entropy computation makes the resulting uncertainty estimates brittle under limited sampling budgets.

In this work, we propose a principled information-theoretic framework for semantic uncertainty estimation in LLMs that overcomes these limitations by combining soft semantic community discovery with kernel Rényi entropy [16,17]. Instead of enforcing hard semantic equivalence classes, we represent sampled generations as nodes in a weighted semantic graph and infer soft community memberships via weighted graph community detection [18,19]. These soft assignments naturally capture overlapping and ambiguous semantic relationships while avoiding the order dependence of greedy clustering.

The inferred communities induce a positive semi-definite semantic kernel whose spectrum characterizes the distribution of semantic modes expressed by the LLM. We quantify uncertainty by computing the Rényi entropy of the kernel spectrum, yielding a tunable uncertainty measure that interpolates between sensitivity to dominant semantic interpretations and robustness to rare or noisy semantic variations. This spectral perspective decouples semantic structure discovery from uncertainty quantification and generalizes existing entropy-based estimators as a special case.

Through extensive experiments on question-answering tasks, we demonstrate that the proposed framework produces more stable, discriminative, and sample-efficient uncertainty estimates compared to prior semantic entropy methods. Our results highlight the advantages of soft community modeling and Rényi spectral analysis for semantic uncertainty quantification in LLMs.

The main contributions of this work are summarized as follows:

- We propose a soft-community formulation for semantic uncertainty estimation in LLMs, representing multiple sampled generations as a weighted semantic graph and inferring soft community memberships instead of enforcing hard semantic equivalence classes.
- We introduce a kernel-based Rényi spectral uncertainty estimator, which quantifies semantic uncertainty via the Rényi entropy of the semantic kernel spectrum, generalizing von Neumann entropy and enabling tunable sensitivity to dominant and long-tail semantic modes.
- We present a unified information-theoretic framework that decouples semantic structure discovery from uncertainty quantification, providing a principled and extensible perspective on semantic uncertainty estimation for LLMs.
- Extensive experiments demonstrate that the proposed method yields more stable, discriminative, and sample-efficient uncertainty estimates under limited sampling budgets and noisy semantic judgments compared to existing semantic entropy approaches.

## 2. Related Work

### 2.1. Uncertainty Estimation in Large Language Models

Uncertainty estimation is a fundamental component in assessing the reliability of large language model (LLM) outputs, particularly for hallucination detection and risk-aware deployment. Early approaches primarily relied on token-level predictive distributions, such as entropy or variance computed from output probabilities. While effective for capturing lexical uncertainty, these measures often fail to reflect ambiguity at the semantic level, where responses may differ in meaning despite similar surface forms [8,9].

Motivated by this limitation, recent work has increasingly focused on semantic uncertainty estimation, which characterizes uncertainty over sets of sampled responses rather than individual tokens. In this paradigm, uncertainty reflects the diversity of semantic interpretations produced by an LLM for a given query and has been shown to correlate more closely with hallucination likelihood [10]. A common strategy is to evaluate semantic similarity between sampled responses using natural language inference (NLI) models or sentence embeddings, followed by aggregation of these similarities into a global uncertainty measure [20].

Several black-box semantic uncertainty estimators adopt graph-based formulations, where sampled responses are represented as nodes and edge weights encode semantic similarity. Uncertainty is then inferred from structural properties of the resulting graph. For example, some methods estimate uncertainty via node degree statistics, eccentricity measures, or spectral characteristics of the graph Laplacian [20]. While these approaches provide flexible representations of semantic relationships, they often rely on specific graph proxies whose sensitivity to semantic noise and sampling variability may be difficult to control.

Beyond structural graph measures, entropy-based semantic uncertainty estimators have been proposed to directly quantify the dispersion of semantic interpretations. Semantic entropy [10] computes uncertainty by clustering responses into semantic equivalence classes and applying Shannon entropy to the resulting distribution. Subsequent extensions have explored finer-grained semantic modeling, including discrete semantic units [11], equivalence-aware decompositions [21], and smooth uncertainty estimation based on transformer-derived sentence embeddings [22]. These methods highlight the benefits of moving beyond strict entailment decisions, but many still rely on hard clustering assumptions or fixed entropy formulations.

Graph- and kernel-based perspectives further generalize semantic uncertainty estimation by embedding semantic relationships into positive semi-definite matrices and analyzing their spectral properties [23]. Von Neumann entropy has been employed to summarize semantic dispersion via the normalized kernel spectrum [24]. However, this formulation corresponds to a fixed Shannon entropy over eigenvalues and offers limited flexibility in controlling the contribution of dominant versus long-tail semantic modes. Moreover, hard equivalence assumptions or rigid structural proxies may still limit robustness under noisy semantic judgments. Taken together, these methods suggest that semantic uncertainty can be viewed as a spectral property of semantic similarity structures, rather than a by-product of token-level confidence.

In parallel, there is growing interest in uncertainty estimation for long-form and open-ended generation tasks, where responses may span multiple sentences or paragraphs and exhibit richer semantic variation [25,26,27]. In contrast, the present work focuses on short-form, proposition-level responses, where uncertainty arises primarily from competing semantic interpretations rather than extended discourse structure [10]. This setting provides a controlled testbed for studying semantic uncertainty and evaluating the robustness of uncertainty estimators.

### 2.2. Hallucinations and Confabulations in Large Language Models

Hallucinations in LLMs broadly refer to the generation of fluent but unsupported, incorrect, or internally inconsistent content. Prior studies have identified multiple manifestations of hallucination, including factual fabrication, instruction inconsistency, and reasoning failures, across tasks such as question answering, summarization, and code generation [10,28,29,30]. These phenomena pose a significant challenge to the reliable deployment of LLMs, particularly in scenarios requiring factual accuracy and logical consistency.

Within this broad category, a specific and practically important form of hallucination is often referred to as confabulation, also described as fabrication in short-form question-answering settings [10]. Confabulation occurs when an LLM produces an answer despite lacking sufficient knowledge to support it, leading to responses that are arbitrary or mutually inconsistent across repeated generations. For example, when presented with the same factual query, a model may output different, incompatible answers in separate samples, indicating the absence of a stable underlying semantic belief.

A commonly cited explanation for confabulation is the tendency of LLMs to generate a response even when a query exceeds their effective knowledge boundaries. This behavior is closely tied to training objectives that reward fluent answer generation rather than calibrated abstention, resulting in an overconfident or over-eager response pattern [10,28]. As a consequence, models may prefer to produce a plausible-sounding answer instead of signaling uncertainty or deferring the question.

A variety of approaches have been proposed to mitigate or detect hallucinations and confabulations. Some methods rely on external knowledge sources, cross-referencing generated content with curated databases or retrieval systems to verify factual correctness [31]. Other approaches employ auxiliary models, such as using an external LLM as a judge to assess the consistency or plausibility of generated responses [32]. While effective in certain settings, these strategies typically require additional resources, supervision, or task-specific infrastructure.

An alternative line of research formulates hallucination detection as a supervised classification problem, training models to distinguish accurate from fabricated content using internal representations of the LLM [33]. Although promising, such methods depend on labeled data and may struggle to generalize across domains or prompt distributions.

More recently, uncertainty-based approaches have gained attention as unsupervised indicators of hallucination risk. In particular, semantic uncertainty has been shown to correlate strongly with confabulation in short-form question answering, where competing semantic interpretations emerge across multiple sampled responses [10]. In such settings, uncertainty estimation provides a lightweight and task-agnostic proxy for identifying hallucination-prone queries.

Despite these advances, the effectiveness of uncertainty-based hallucination detection critically depends on the robustness of the underlying uncertainty estimator. Sensitivity to clustering heuristics, rigid equivalence assumptions, or fixed entropy formulations can limit reliability under noisy semantic judgments. This highlights the need for more flexible and principled semantic uncertainty frameworks that can better support the detection of hallucinations and confabulations in large language models.

## 3. Method

### 3.1. Problem Setup

Let *x* denote an input query, such as a short-form factual question. Given a large language model M and a sampling strategy (e.g., temperature sampling), we generate a set of *N* responses

(1) $$S\left(x\right)=\left\{s_{1},s_{2},\ldots ,s_{N}\right\},$$

where each $s_{i}$ represents a complete natural language response sampled independently from M conditioned on *x*.

Our goal is to quantify the semantic uncertainty of M with respect to *x*, defined as the degree of disagreement among the semantic interpretations expressed by the sampled responses. Unlike token-level uncertainty measures, which operate on predictive distributions over vocabulary items, semantic uncertainty is defined over the space of meanings induced by S(x).

**Figure 1:**
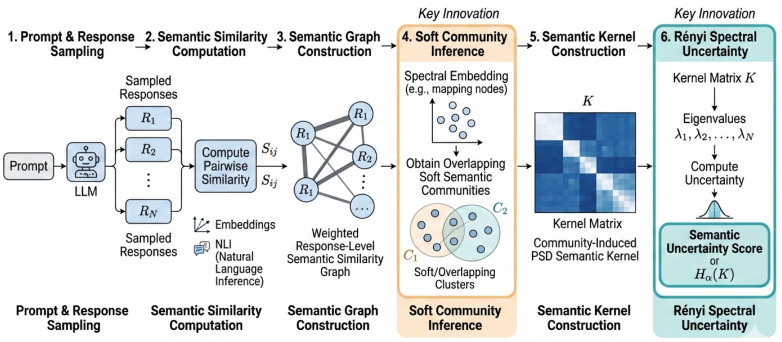
Overview of the proposed RSU framework for semantic uncertainty estimation in large language models. Given an input prompt, the language model first samples multiple candidate responses. Pairwise semantic similarities between responses are then computed by combining embedding-based similarity and natural language inference (NLI) signals, and used to construct a weighted response-level semantic similarity graph. Based on this graph, the proposed method performs spectral embedding and infers overlapping soft semantic communities, rather than relying on hard semantic clustering. These soft communities are subsequently used to construct a community-induced positive semi-definite semantic kernel matrix *K*. Finally, the eigenvalue spectrum of *K* is analyzed through Rényi entropy to produce a query-level semantic uncertainty score $H_{\alpha }\left(K\right)$. The two key innovations of the framework are the soft community inference module and the Rényi spectral uncertainty estimation module, which together decouple semantic structure discovery from uncertainty quantification.

Figure 1 provides a step-by-step overview of the proposed semantic uncertainty estimation framework, illustrating how sampled responses are organized into soft semantic communities and summarized via Rényi spectral entropy. We next formalize each component of the framework. Throughout the paper, we focus on query-level semantic uncertainty estimation and do not assume access to model internals.

While several ingredients of the framework, such as semantic similarity graphs, sentence embedding/NLI-based similarity estimation, and spectral graph analysis, are adapted from existing literature, they are not the primary novelty of this work. The main methodological contribution lies in their integration into a unified semantic uncertainty framework based on soft community inference and Rényi spectral kernel entropy. In particular, the proposed method replaces hard semantic equivalence classes with soft community assignments, constructs a community-induced positive semi-definite semantic kernel, and quantifies uncertainty through the Rényi entropy of its spectrum. This design decouples semantic structure discovery from uncertainty quantification and generalizes existing Shannon- or von Neumann-style formulations.

### 3.2. Semantic Similarity and Graph Construction

To model semantic relationships among sampled responses, we represent S(x) as a weighted undirected graph

(2) G=(V,E,W),

where each node $v_{i}\in V$ corresponds to a response $s_{i}$, and edge weights $W_{ij}\in \left[0,1\right]$ encode the semantic similarity between responses $s_{i}$ and $s_{j}$.

We compute semantic similarity by jointly leveraging sentence-level semantic embeddings and NLI scores, which capture complementary aspects of semantic relatedness [13,34,35]. Specifically, sentence embeddings provide a continuous notion of global semantic proximity, while NLI scores capture directional, logic-aware semantic entailment. NLI is a fundamental task in natural language understanding that aims to determine the semantic relationship between a premise and a hypothesis, typically categorized as entailment, contradiction, or neutrality. In the context of semantic uncertainty estimation, NLI provides a logic-aware measure of semantic consistency that goes beyond surface-level semantic similarity. While sentence embeddings primarily capture distributional or topical proximity between responses, they may assign high similarity to statements that are semantically related yet factually inconsistent. In contrast, NLI explicitly models whether one response semantically supports another, making it particularly suitable for detecting factual disagreement and mutual inconsistency among short-form generated answers.

Let $e\left(s_{i}\right)\in R^{d}$ denote the fixed sentence embedding of response $s_{i}$, obtained from a pretrained sentence embedding model (e.g., all-mpnet-base-v2 [14]) that is independent of the language model used for response generation. We first compute an embedding-based similarity

(3) $$S_{ij}^{\mathrm{emb}}=\frac{\langle e\left(s_{i}\right),e\left(s_{j}\right)\rangle }{∥e\left(s_{i}\right)∥\;∥e\left(s_{j}\right)∥},$$

which is then linearly rescaled to [0,1] for numerical consistency.

In parallel, we compute a symmetric NLI-based similarity score

(4) $$S_{ij}^{\mathrm{nli}}=\sigma \;\left(\mathrm{NLI}(s_{i}\Rightarrow s_{j})\right)\cdot \sigma \;\left(\mathrm{NLI}(s_{j}\Rightarrow s_{i})\right),$$

where $\mathrm{NLI}(s_{i}\Rightarrow s_{j})$ denotes the entailment score from $s_{i}$ to $s_{j}$, and σ(·) denotes the Sigmoid activation function that maps raw scores to [0,1]. This formulation captures mutual semantic support while remaining agnostic to strict equivalence decisions.

We combine the two similarity measures through a multiplicative fusion:

(5) $$W_{ij}={\left(S_{ij}^{\mathrm{emb}}\right)}^{\eta }\cdot {\left(S_{ij}^{\mathrm{nli}}\right)}^{1-\eta },\;\eta \in \left[0,1\right],$$

which yields the final edge weight in the semantic graph. This fusion emphasizes response pairs that are both semantically close in embedding space and mutually entailed under NLI, while suppressing spurious similarity arising from either measure alone. The fusion parameter *η* plays a role analogous to a temperature that balances geometric and logical notions of semantic similarity. In our experiment, we fix *η* = 0.5 and do not tune it per dataset. An ablation study on the effect of the fusion weight *η* is provided in Section 4.5.2.

### 3.3. Soft Community Representation

Rather than enforcing hard semantic equivalence classes, we infer a soft community structure over the semantic graph to capture graded and overlapping semantic relationships among sampled responses. Specifically, we estimate a community assignment matrix

(6) $$P\in R^{N\times K},$$

where $P_{ik}\geq 0$ denotes the membership strength of response $s_{i}$ in semantic community *k*, and $\sum_{k=1}^{K}P_{ik}=1$ for all *i*. In practice, *K* corresponds to the number of leading non-trivial spectral components used in the graph embedding and is treated as a fixed hyperparameter across datasets.

To compute the soft community assignment matrix *P*, we first perform a spectral analysis of the semantic similarity graph. Let $W\in R^{N\times N}$ denote the semantic similarity matrix and *D* the corresponding degree matrix with $D_{ii}=\sum_{j}W_{ij}$. We construct the symmetric normalized graph Laplacian

(7) $$L_{\mathrm{sym}}=I-D^{-1/2}WD^{-1/2},$$

which is positive semi-definite and admits an orthogonal eigendecomposition.

We compute the eigenpairs of $L_{\mathrm{sym}}$,

(8) $$L_{\mathrm{sym}}u_{k}=\lambda_{k}u_{k},$$

with eigenvalues ordered as $0=\lambda_{1}\leq \lambda_{2}\leq \ldots \leq \lambda_{N}$. A spectral embedding is then formed by stacking the eigenvectors corresponding to the smallest non-trivial eigenvalues:

(9) $$U=\left[u_{2},u_{3},\ldots ,u_{K+1}\right]\in R^{N\times K}.$$

Each row of *U* provides a low-dimensional semantic representation of a sampled response. The first eigenvector $u_{1}$ is excluded, as it corresponds to a trivial uniform mode associated with the zero eigenvalue and does not encode discriminative semantic structure.

Given the spectral embedding *U*, we obtain soft community memberships via a row-wise softmax operation:

(10) $$P_{ik}=\frac{\exp(\tau U_{ik})}{\sum_{k^{\prime }=1}^{K}\exp\left(\tau U_{ik^{\prime }}\right)},$$

where $U_{ik}$ denotes the (i,k)-th element of *U* and τ>0 controls the sharpness of the assignment. This probabilistic formulation yields overlapping communities and avoids the order sensitivity and instability of hard clustering procedures.

Finally, each semantic community is represented by a weighted aggregate embedding

(11) $$c_{k}=\sum_{i=1}^{N}P_{ik}\;e\left(s_{i}\right),$$

where $e(s_{i})$ denotes the sentence-level semantic embedding of response $s_{i}$. This representation summarizes each community as a soft semantic prototype and serves as the basis for subsequent kernel construction and spectral uncertainty estimation.

### 3.4. Semantic Kernel and Rényi Spectral Uncertainty

Given the soft community representations ${\left\{c_{k}\right\}}_{k=1}^{K}$ obtained from the semantic graph, we construct a kernel-based representation that summarizes the global semantic structure of the sampled responses. Specifically, we define a positive semi-definite semantic kernel

(12) $$G=\sum_{k=1}^{K}\omega_{k}\;c_{k}c_{k}^{⊤},$$

where

(13) $$\omega_{k}=\frac{1}{N}\sum_{i=1}^{N}P_{ik}$$

denotes the relative prevalence of semantic community *k* among the sampled responses. This formulation aggregates community-level semantic prototypes while weighting them according to their empirical support, yielding a compact second-order representation of semantic structure. Here, each community prototype $c_{k}\in R^{d}$ is a sentence embedding-level representation, and the resulting semantic kernel $G\in R^{d\times d}$ captures second-order semantic structure in the embedding space.

The semantic kernel G captures both the diversity and dominance of semantic communities in a unified matrix form that is amenable to spectral analysis. To ensure numerical stability and comparability across different queries, we normalize the kernel to unit trace:

(14) $$\tilde{G}=\frac{G}{\mathrm{Tr}(G)}.$$

The resulting normalized kernel can be interpreted as a distribution over semantic modes, with its eigenvalues reflecting the relative importance of distinct semantic directions.

We quantify semantic uncertainty by computing the Rényi entropy of the spectrum of $\tilde{G}$. Let $\left\{\lambda_{1},\ldots ,\lambda_{d}\right\}$ denote the eigenvalues of the normalized kernel $\tilde{G}$. The Rényi semantic uncertainty of order α>0, α≠1 is defined as

(15) $$U_{\alpha }\left(x\right)=\frac{1}{1-\alpha }\log\sum_{i=1}^{d}\lambda_{i}^{\alpha }.$$

The order parameter α controls the sensitivity of the uncertainty measure to the kernel spectrum. Larger values of α emphasize dominant semantic modes, corresponding to widely shared interpretations, whereas smaller values increase sensitivity to long-tail semantic variation that may arise from minority or unstable interpretations. In the limit α→1, the Rényi semantic uncertainty recovers the Shannon (von Neumann) entropy as a special case.

This kernel-based spectral formulation decouples semantic structure discovery from uncertainty quantification and provides a flexible information-theoretic framework for measuring semantic disagreement among sampled responses. The complete procedure is summarized in Algorithm 1.
**Algorithm 1:** Soft-Community Rényi Semantic Uncertainty**Input**:Query *x*, language model M, number of samples *N*, Rényi order α, fusion weight η∈[0,1], soft assignment temperature τ>0, number of communities *K***Output**:Semantic uncertainty score $U_{\alpha }\left(x\right)$**Sampling.** Sample *N* responses $S\left(x\right)=\left\{s_{1},\ldots ,s_{N}\right\}$ from M conditioned on *x*;**Semantic similarity.** Obtain sentence embeddings $e(s_{i})$ for each response. Compute embedding similarity $S_{ij}^{\mathrm{emb}}$ (rescaled to [0,1]) and symmetric NLI similarity $S_{ij}^{\mathrm{nli}}=\sigma \left(\mathrm{NLI}\left(s_{i}\Rightarrow s_{j}\right)\right)\cdot \sigma \left(\mathrm{NLI}\left(s_{j}\Rightarrow s_{i}\right)\right)$. Fuse similarities to form the graph weights $W_{ij}={\left(S_{ij}^{\mathrm{emb}}\right)}^{\eta }{\left(S_{ij}^{\mathrm{nli}}\right)}^{1-\eta }$;**Spectral embedding.** Compute degree matrix *D* with $D_{ii}=\sum_{j}W_{ij}$ and normalized Laplacian $L_{\mathrm{sym}}=I-D^{-1/2}WD^{-1/2}$. Compute eigenpairs $L_{\mathrm{sym}}u_{k}=\lambda_{k}u_{k}$ and form $U=\left[u_{2},\ldots ,u_{K+1}\right]\in R^{N\times K}$;**Soft communities.** Compute soft memberships by row-wise softmax: $P_{ik}=\exp\left(\tau U_{ik}\right)/\sum_{k^{\prime }=1}^{K}\exp\left(\tau U_{ik^{\prime }}\right)$;**Kernel construction.** Compute community prototypes $c_{k}=\sum_{i=1}^{N}P_{ik}e\left(s_{i}\right)$ and weights $\omega_{k}=\frac{1}{N}\sum_{i=1}^{N}P_{ik}$. Construct kernel $G=\sum_{k=1}^{K}\omega_{k}\;c_{k}c_{k}^{⊤}$ and normalize $\tilde{G}=G/\mathrm{Tr}\left(G\right)$;**Rényi spectral uncertainty.** Compute eigenvalues $\left\{\lambda_{i}\right\}$ of $\tilde{G}$ and output$$U_{\alpha }\left(x\right)=\frac{1}{1-\alpha }\log\sum_{i}\lambda_{i}^{\alpha }.$$**return** $U_{\alpha }\left(x\right)$;

We next analyze the computational complexity of the proposed approach and compare it with the original hard-clustering semantic entropy framework. Let *N* denote the number of sampled responses, *d* the dimensionality of the sentence embeddings, and *K* the number of semantic communities.

Computing pairwise semantic similarity scores, including both embedding-based and NLI-based components, requires $O(N^{2})$ operations. We construct the normalized graph Laplacian and perform eigendecomposition to obtain the spectral embedding for soft community inference scales as $O(N^{3})$ in the worst case. Compared with the original hard-clustering pipeline, this step introduces additional overhead, but the cost remains modest in practice due to the small number of sampled responses.

Given the spectral embedding, computing soft community assignments via a row-wise softmax requires O(NK) operations. Constructing the semantic kernel incurs $O(Kd^{2})$ cost, and computing the Rényi spectral uncertainty involves eigendecomposition of a d×d kernel matrix, which scales as $O(d^{3})$ in the worst case. This kernel-level spectral computation also constitutes additional cost relative to Shannon-entropy-based semantic entropy, but enables a richer uncertainty characterization through the proposed spectral Rényi formulation.

Overall, the computational complexity is dominated by the eigendecomposition steps on the response-level graph and the embedding-level kernel. In practice, since *N* is small (typically on the order of tens) and *d* is moderate and fixed by the embedding model, the overall computation remains efficient and tractable for query-level semantic uncertainty estimation. Therefore, the additional computation can be viewed as a reasonable trade-off for improved robustness, yielding uncertainty estimates that are less sensitive to clustering order, semantic ambiguity, and noisy pairwise relations.

### 3.5. Theoretical Properties of Rényi Spectral Uncertainty

We analyze several fundamental properties of the proposed Rényi spectral uncertainty measure. Let $\tilde{G}$ be a unit-trace positive semi-definite semantic kernel with eigenvalues ${\left\{\lambda_{i}\right\}}_{i=1}^{d}$. For α>0, α≠1, the Rényi semantic uncertainty is defined as

(16) $$U_{\alpha }\left(\tilde{G}\right)=\frac{1}{1-\alpha }\log\sum_{i=1}^{d}\lambda_{i}^{\alpha }.$$

**Proposition** **1** (Degeneracy). *If $\tilde{G}$ has rank one, i.e., $\lambda_{1}=1$ and $\lambda_{i}=0$ for all i>1, then $U_{\alpha }\left(\tilde{G}\right)=0$ for any α>0.*

**Proof.**  All proofs are provided in Appendix A. □

**Proposition** **2** (Unitary Invariance). *For any orthogonal matrix U, the Rényi spectral uncertainty is invariant under orthogonal similarity transformations:*

(17) $$U_{\alpha }\left(\tilde{G}\right)=U_{\alpha }\left(U\tilde{G}U^{⊤}\right).$$

**Proposition** **3** (Monotonicity under Spectral Dispersion). *For fixed trace, $U_{\alpha }\left(\tilde{G}\right)$ increases as the eigenvalue distribution of $\tilde{G}$ becomes more uniform. In particular, kernels with more evenly distributed eigenvalues exhibit higher semantic uncertainty.*

**Proposition** **4** (Order Sensitivity). *The Rényi order α controls the sensitivity of $U_{\alpha }$ to dominant semantic modes. Larger values of α emphasize large eigenvalues, making the uncertainty measure more sensitive to dominant semantic interpretations, whereas smaller values of α increase sensitivity to long-tail semantic variation.*

Together, these properties characterize the behavior of the proposed semantic uncertainty measure. Proposition 1 ensures that uncertainty vanishes when all sampled responses collapse to a single semantic interpretation, corresponding to maximal semantic certainty. Proposition 2 guarantees that the uncertainty depends only on the intrinsic spectral structure of the semantic kernel and is invariant to the choice of basis or representation.

Proposition 3 formalizes the intuition that semantic uncertainty reflects the dispersion of competing semantic modes: concentration of semantic mass on a small number of dominant modes leads to low uncertainty, whereas a more uniform distribution across modes yields higher uncertainty. Finally, Proposition 4 highlights a key advantage of the Rényi formulation, namely the ability to explicitly control the relative contribution of dominant versus minor semantic interpretations through the order parameter α. This flexibility is particularly valuable under limited sampling, where small eigenvalues may correspond either to noise or to meaningful but infrequent semantic alternatives.

## 4. Experiments

### 4.1. Experimental Setup

We name our approach Rényi spectral uncertainty (RSU), a kernel-based framework for semantic uncertainty estimation in large language models. We evaluate its performance in a black-box question-answering setting. For each input query *x*, we sample multiple independent responses from a pretrained instruction-tuned language model to induce semantic variability. Unless otherwise specified, all experiments are conducted using a fixed sampling budget per query and identical decoding configurations across methods.

Sentence-level semantic embeddings are computed using a fixed pretrained sentence embedding model, which is independent of the language model used for generation. Logical semantic relations are estimated using a pretrained natural language inference (NLI) model. Both models are held fixed throughout all experiments and are not fine-tuned.

For semantic similarity construction, we employ the multiplicative fusion of embedding-based similarity and symmetric NLI entailment scores described in Section 3, with fusion weight *η* controlling their relative contributions. Soft community assignments are obtained via spectral embedding of the normalized graph Laplacian followed by a row-wise softmax with temperature *τ*. The number of communities *K*, the Rényi order *α*, and all other hyperparameters are fixed across datasets unless explicitly varied in ablation studies.

For each input query, we sample *N* = 10 responses using a combination of top-*K* sampling (*K* = 50) and nucleus sampling (*p* = 0.9) at temperature *T* = 1. This setting provides a balance between semantic diversity and generation stability and is consistent with our sensitivity analysis in Section 4.6.

### 4.2. Evaluation Tasks and Data

We consider short-form generative question-answering tasks for evaluation, where semantic uncertainty arises primarily from competing interpretations or incomplete knowledge rather than long-form discourse. This setting provides a controlled environment for analyzing semantic disagreement across sampled responses and its relationship to hallucination and confabulation.

Experiments are conducted on a diverse collection of open-domain and domain-specific question-answering benchmarks, covering conversational, closed-book, and specialized knowledge settings. Specifically, we evaluate on the open-book conversational QA dataset CoQA [36], the closed-book QA dataset TriviaQA [37], the biomedical QA dataset BioASQ [38], and the Natural Questions (NQ) benchmark [39]. These datasets span a wide range of domains and question styles, enabling a comprehensive evaluation of semantic uncertainty estimation under varying knowledge and reasoning requirements.

Following standard practice, we use the development split of CoQA, the deduplicated validation split of TriviaQA (rc.nocontext subset), the validation split of NQ, and the training split of BioASQ. Across all datasets, uncertainty is evaluated at the query level by aggregating information from multiple sampled responses.

For each query, we assess whether uncertainty estimates can reliably distinguish between correct and incorrect model outputs. Correctness labels are obtained using dataset-provided ground-truth answers combined with automated verification procedures, following standard practices in prior uncertainty estimation work.

We utilize four widely adopted off-the-shelf instruction-tuned large language models for evaluation, with model sizes ranging from 1B to 12B parameters. These models include Llama-3.2-1B (https://huggingface.co/meta-llama/Llama-3.2-1B-Instruct, accessed on 1 January 2026), Llama-3.1-8B (https://huggingface.co/meta-llama/Llama-3.1-8B-Instruct, accessed on 1 January 2026), Mistral-7B-v0.3 (https://huggingface.co/mistralai/Mistral-7B-Instruct-v0.3, accessed on 5 January 2026), and Mistral-Nemo-12B (https://huggingface.co/mistralai/Mistral-Nemo-Instruct-2407, accessed on 10 January 2026), allowing us to assess the robustness of the proposed uncertainty estimator across different model scales and architectures.

### 4.3. Evaluation Metrics and Baselines

#### 4.3.1. Evaluation Metrics

We evaluate uncertainty estimates using two complementary metrics. First, we report the area under the receiver operating characteristic curve (AUROC), which measures how well uncertainty scores distinguish between correct and incorrect model outputs. An AUROC of 0.5 corresponds to random discrimination, whereas higher values indicate stronger alignment between uncertainty and answer correctness.

Second, we report the area under the accuracy–rejection curve (AUARC), which quantifies the potential accuracy improvement obtained by rejecting answers with high estimated uncertainty. This metric captures the practical utility of uncertainty estimates in risk-aware deployment scenarios.

#### 4.3.2. Baselines

We compare the proposed Rényi spectral uncertainty estimator with a diverse set of representative uncertainty estimation baselines that reflect different modeling assumptions about semantic variability in generated responses. Rather than focusing on individual implementations, we group these baselines according to their underlying principles for quantifying semantic uncertainty.

The first category consists of semantic entropy-based methods, which cluster sampled responses into semantic equivalence classes and compute uncertainty as the entropy of the resulting empirical distribution. These approaches include semantic entropy (SE) [10], Discrete Semantic Entropy (DSE) [10], and Kernel Language Entropy (KLE) [11], which typically rely on hard clustering decisions and treat semantic interpretations as mutually exclusive.

The second category consists of graph-based semantic uncertainty methods, including Ecc [20], EigV [20], Deg [20], and D-UE [21]. These methods construct a semantic similarity graph over sampled responses and derive uncertainty from structural or spectral properties of the graph, such as node centrality, degree statistics, or eigenvalue-based measures.

We additionally include other state-of-the-art approaches, including Number of Semantic Sets (NSS) [24] and Semantic Embedding Uncertainty (SEU) [22]. All baselines are evaluated using the same sampled response and semantic similarity representations to ensure fair comparison.

### 4.4. Main Results

Table 1 and Table 2 report the AUROC and AUARC performance of different uncertainty estimation methods across a diverse set of model–dataset combinations. Overall, the proposed RSU achieves consistently strong performance and outperforms existing baselines in the majority of settings.

#### 4.4.1. AUROC Performance

Across most model–dataset pairs, RSU attains the highest or second-highest AUROC, indicating improved discrimination between correct and incorrect responses based on uncertainty estimates. Performance gains are particularly evident on datasets where semantic ambiguity or confabulation is more prevalent, such as open-domain and knowledge-intensive question-answering benchmarks. These results suggest that explicitly modeling semantic disagreement at the community level provides a more reliable signal of answer correctness than token-level uncertainty or hard semantic clustering.

Compared to entropy-based baselines that rely on hard semantic equivalence assumptions, RSU benefits from soft community representations that capture graded and overlapping semantic interpretations. Relative to graph-based baselines that exploit response-level relational structure, RSU further improves discrimination by combining probabilistic community memberships with spectral aggregation.

#### 4.4.2. AUARC Performance

As shown in Table 2, RSU also yields consistent improvements in accuracy–rejection performance. By selectively rejecting responses with high estimated uncertainty, the proposed method achieves higher retained accuracy across a wide range of rejection thresholds. This demonstrates that RSU provides uncertainty estimates that are not only discriminative but also practically useful for risk-aware decision making.

Across language models of varying sizes and architectures, RSU exhibits robust behavior, indicating that its effectiveness does not depend on specific model internals. Instead, performance gains stem from the explicit modeling of semantic structure among sampled responses, reinforcing the applicability of RSU in black-box settings.

Across all evaluated models and datasets, RSU consistently achieves the best or second-best performance, with only moderate variance across runs. Taken together, these results demonstrate that the proposed framework offers a robust and effective measure of semantic uncertainty, generalizing existing entropy- and graph-based approaches while providing improved discrimination and practical utility.

### 4.5. Ablation Studies

We further conduct a series of ablation studies to analyze the contribution of key design choices in RSU and to better understand why the proposed framework yields robust semantic uncertainty estimates. All ablations are performed using the same evaluation protocol as in the main experiments, while varying one factor at a time.

#### 4.5.1. Effect of Rényi Order α

We first study the effect of the Rényi order *α*, which controls the spectral sensitivity of the proposed RSU. Recall that smaller values of *α* emphasize low-magnitude eigenvalues corresponding to long-tail or rare semantic variations, whereas larger values of *α* increasingly focus on dominant semantic modes.

We conduct this ablation on Llama-3.2-1B across all four datasets, varying α∈{0.2,0.5,1,2,5,10} while keeping all other components fixed. The Shannon (von Neumann) entropy case is approximated using *α* = 1.01 for numerical stability.

Table 3 reports the AUROC results. Across all datasets, RSU exhibits a clear and consistent U-shaped performance profile as a function of *α*. Very small values of *α* lead to degraded performance, as the uncertainty measure becomes overly sensitive to minor eigenvalues that often reflect sampling noise or spurious semantic variations. As *α* increases, performance improves steadily and peaks around *α* = 2, indicating an optimal balance between dominant and secondary semantic interpretations.

For larger values of *α* (e.g., *α* = 5 and *α* = 10), performance slightly decreases, suggesting that over-emphasizing the dominant spectral components suppresses meaningful semantic disagreement. Importantly, this trend is consistent across all datasets, including open-domain (NQ, TriviaQA) and knowledge-intensive (BioASQ) benchmarks. This observation is consistent with the theoretical role of *α* as a control over spectral sensitivity, as discussed in Section 3.4.

Based on these observations, we adopt *α* = 2 as the default setting in all main experiments. This choice is empirically robust and aligns with the theoretical motivation that effective semantic uncertainty estimation requires balancing dominant interpretations against structured semantic diversity.

#### 4.5.2. Effect of Fusion Weight η

RSU constructs the semantic similarity graph by fusing two complementary signals: sentence embedding similarity and entailment-based semantic consistency. The fusion weight η∈[0,1] controls their relative contributions, where η→0 yields a graph dominated by entailment-based similarity, and η→1 relies primarily on embedding-based similarity.

We evaluate the effect of *η* on Llama-3.2-1B across all datasets by varying η∈{0.01,0.25,0.5,0.75,0.99}, while keeping all other components fixed. The corresponding AUROC results are reported in Table 4.

Overall, intermediate values of *η* consistently achieve the best performance, with *η* = 0.5 yielding the highest average AUROC. When *η* approaches either extreme, performance degrades, indicating that relying exclusively on a single similarity source is suboptimal. In particular, graphs constructed solely from embedding-based similarity (η→1) exhibit the largest performance drop. This behavior can be attributed to the fact that sentence embeddings primarily capture distributional and topical similarity, which may conflate semantically related but factually inconsistent answers. As a result, embedding-only graphs tend to underestimate semantic disagreement in short-form question answering, leading to overconfident uncertainty estimates.

In contrast, entailment-only graphs (η→0) perform more competitively, as entailment models are explicitly trained to detect logical consistency and contradiction, making them more sensitive to factual conflicts among sampled responses. However, entailment predictions are inherently noisy and discrete, which may introduce instability in the induced graph structure and subsequent spectral analysis.

The best performance is achieved by fusing the two signals. Embedding-based similarity provides a smooth geometric structure that stabilizes the semantic graph, while entailment-based similarity supplies strong discriminative cues for logical inconsistency. Their combination allows RSU to capture both continuous semantic proximity and discrete logical disagreement, resulting in a more robust and expressive semantic kernel for uncertainty estimation.

#### 4.5.3. Soft vs. Hard Semantic Community Assignment

We further investigate the impact of soft semantic community modeling by comparing RSU with a hard community variant. In the hard assignment setting, each response is assigned to a single semantic community by selecting the community with the highest spectral embedding activation. Formally, given the spectral embedding $U\in R^{N\times K}$, hard assignments are obtained via $P_{ik}=1\left[k=\arg\max_{j}U_{ij}\right]$, resulting in mutually exclusive semantic communities.

Table 5 reports the AUROC comparison. Across all datasets, soft community assignment consistently outperforms hard clustering. This performance gap highlights the importance of modeling graded and overlapping semantic relationships among sampled responses.

Hard community assignment enforces strict semantic boundaries, which can be problematic in short-form question answering where responses may partially agree, share common entities, or differ only in subtle factual details. Such rigid partitioning may artificially fragment semantically related answers or collapse distinct but related interpretations, leading to distorted semantic structure.

In contrast, soft community modeling allows each response to contribute to multiple semantic communities with different strengths. This probabilistic representation preserves nuanced semantic overlap and yields a smoother and more stable semantic kernel. As a result, the subsequent spectral analysis captures semantic disagreement more faithfully, leading to improved uncertainty estimation.

### 4.6. Sensitivity Analysis

We finally analyze the sensitivity of RSU with respect to the number of sampled responses *N*. RSU estimates semantic uncertainty by aggregating information across multiple sampled responses. We therefore examine the sensitivity of the proposed method to the number of samples *N*. Specifically, we vary N∈{3,5,10,20,50} while keeping all other components fixed.

Table 6 reports the AUROC results across all datasets. When the number of samples is very small (*N* = 3), performance is substantially degraded, indicating that insufficient sampling fails to capture the underlying semantic structure. As *N* increases, performance improves rapidly and stabilizes around *N* = 10.

Notably, further increasing the number of samples beyond *N* = 10 yields only marginal improvements. This saturation behavior suggests that RSU is able to recover reliable semantic uncertainty estimates with a relatively small number of samples, making it computationally efficient in practice. Across all datasets, the relative performance trends remain consistent, further confirming the robustness of the proposed method with respect to sampling size.

## 5. Conclusions and Future Work

This work adopts a spectral, information-theoretic perspective on semantic uncertainty estimation in large language models. A central design choice is the use of Rényi entropy over the spectrum of a semantic kernel, which generalizes existing Shannon- and von Neumann-based formulations and defines a parametric family that explicitly controls the relative contribution of dominant versus long-tail spectral components. In the context of semantic uncertainty, this flexibility is crucial: dominant eigenvalues typically correspond to widely shared semantic interpretations, whereas smaller eigenvalues capture minority, unstable, or competing semantic alternatives. The Rényi order *α* therefore provides a principled mechanism for balancing sensitivity to semantic disagreement against robustness under limited and noisy sampling. This spectral formulation is further strengthened by the use of soft, community-aware semantic representations, which model semantic similarity as inherently graded and avoid the noise amplification and order dependence associated with hard semantic equivalence classes.

Building on these design principles, we introduced Rényi spectral uncertainty (RSU), a principled and flexible framework for semantic uncertainty estimation in large language models. By modeling semantic relationships among multiple sampled responses as a weighted graph and adopting a soft community representation, RSU captures graded and overlapping semantic structures beyond hard equivalence assumptions. Extensive experiments across multiple question-answering benchmarks and language models demonstrate that RSU consistently provides stronger discrimination between correct and incorrect responses and yields more effective accuracy–rejection trade-offs than existing uncertainty estimators. These results highlight the importance of integrating semantic structure discovery with information-theoretic uncertainty measures for uncertainty-aware deployment of large language models.

Several directions remain open for future work. While RSU is particularly effective for short-form, proposition-level responses, the overall framework is not restricted to question answering. More generally, it can be applied to other LLM generation tasks, such as summarization, dialogue, retrieval-augmented generation, and long-form text generation, whenever multiple sampled outputs can be interpreted as alternative semantic realizations of the same prompt. In such settings, the core pipeline of semantic graph construction, soft community inference, and Rényi spectral uncertainty estimation remains applicable, while the main challenge lies in designing task-appropriate semantic similarity measures. For instance, long-form generation may require discourse-aware or segment-level semantic comparisons, whereas retrieval-grounded generation may benefit from evidence-aware similarity modeling.

In addition, integrating more adaptive or task-specific semantic similarity models may further enhance robustness across domains, since semantic similarity directly determines the response graph structure and therefore strongly influences the resulting uncertainty estimate. While the current embedding- and NLI-based similarity design works well for the short-form QA setting considered here, other applications may require more specialized modeling, such as domain-adapted sentence encoders, task-specific fusion of different similarity signals, evidence-aware similarity for retrieval-grounded generation, or discourse-aware similarity for long-form generation. Moreover, if supervised signals such as hallucination or factuality labels are available, the proposed framework could be further extended through supervised calibration or learned similarity modeling. For example, one may learn task-specific fusion weights for the semantic graph, replace the fixed similarity components with trainable semantic comparators, or calibrate the resulting Rényi spectral uncertainty score into a downstream hallucination risk predictor.

## Figures and Tables

**Table 1:** Performance (AUROC, %) comparison of uncertainty estimation methods. Results are reported as mean ± standard deviation. For each model–dataset combination, the best performance is highlighted in bold, and the second-best performance is underlined.

Dataset	Entropy-Based Methods			Graph-Based Methods				Consistency-Based Methods		Ours
	SE	DSE	KLE	Ecc	EigV	Deg	D-UE	NSS	SEU	RSU
Llama-3.2-1B										
NQ	77.48 ± 0.55	76.50 ± 0.53	75.36 ± 0.50	76.66 ± 0.62	75.87 ± 0.57	77.18 ± 0.51	71.93 ± 0.63	76.43 ± 0.56	67.38 ± 0.72	**77.90** ± **0.54**
CoQA	73.73 ± 0.21	73.22 ± 0.22	74.59 ± 0.28	73.84 ± 0.29	70.82 ± 0.25	75.73 ± 0.27	73.95 ± 0.28	72.51 ± 0.22	69.80 ± 0.27	**75.75 ± 0.28**
BioASQ	86.87 ± 0.45	86.76 ± 0.47	86.73 ± 0.40	86.79 ± 0.45	85.53 ± 0.42	87.25 ± 0.39	85.62 ± 0.44	86.36 ± 0.44	78.78 ± 0.51	**87.55 ± 0.39**
TriviaQA	82.17 ± 0.18	81.15 ± 0.16	80.41 ± 0.18	81.13 ± 0.18	78.84 ± 0.16	81.64 ± 0.16	79.25 ± 0.13	80.51 ± 0.15	77.04 ± 0.14	**82.23 ± 0.16**
Average	80.06	79.41	79.27	79.61	77.77	80.45	77.69	78.95	73.25	**80.86**
Llama-3.1-8B										
NQ	78.30 ± 0.43	77.88 ± 0.47	77.55 ± 0.44	77.73 ± 0.46	76.26 ± 0.41	78.64 ± 0.44	75.00 ± 0.42	77.48 ± 0.47	71.03 ± 0.44	**78.86 ± 0.43**
CoQA	75.26 ± 0.36	74.89 ± 0.35	78.92 ± 0.27	76.97 ± 0.40	71.75 ± 0.33	80.04 ± 0.24	77.90 ± 0.30	74.14 ± 0.35	72.71 ± 0.33	**80.32 ± 0.27**
BioASQ	83.40 ± 0.47	83.35 ± 0.47	84.28 ± 0.45	83.03 ± 0.58	81.32 ± 0.42	84.73 ± 0.46	82.59 ± 0.57	82.45 ± 0.51	74.81 ± 0.74	**84.92 ± 0.48**
TriviaQA	85.95 ± 0.11	85.23 ± 0.13	85.67 ± 0.12	84.97 ± 0.24	83.27 ± 0.12	86.23 ± 0.12	84.51 ± 0.38	84.42 ± 0.13	81.95 ± 0.13	**87.11 ± 0.13**
Average	80.73	80.34	81.61	80.68	78.15	82.41	80.00	79.62	75.13	**82.80**
Mistral-7B-v0.3										
NQ	76.88 ± 0.60	76.88 ± 0.60	77.58 ± 0.55	77.24 ± 0.46	76.62 ± 0.37	77.42 ± 0.56	76.15 ± 0.45	76.67 ± 0.57	71.85 ± 0.43	**77.79 ± 0.48**
CoQA	75.82 ± 0.33	75.76 ± 0.29	77.60 ± 0.21	78.11 ± 0.35	72.18 ± 0.27	79.61 ± 0.28	78.44 ± 0.26	75.32 ± 0.28	73.47 ± 0.25	**80.21 ± 0.24**
BioASQ	80.86 ± 0.53	80.90 ± 0.50	83.66 ± 0.41	83.05 ± 0.50	82.66 ± 0.50	83.57 ± 0.53	80.54 ± 0.55	80.98 ± 0.49	67.84 ± 0.41	**84.38 ± 0.52**
TriviaQA	83.76 ± 0.29	83.53 ± 0.28	83.86 ± 0.28	83.74 ± 0.11	82.80 ± 0.12	85.04 ± 0.28	83.58 ± 0.28	82.98 ± 0.28	79.59 ± 0.12	**85.22 ± 0.26**
Average	79.33	79.27	80.68	80.54	78.57	81.41	79.68	78.99	73.19	**81.90**
Mistral-Nemo-12B										
NQ	76.78 ± 0.59	76.35 ± 0.57	77.78 ± 0.58	76.55 ± 0.47	76.28 ± 0.58	76.92 ± 0.52	73.04 ± 0.44	75.84 ± 0.56	69.53 ± 0.39	**78.38 ± 0.51**
CoQA	76.08 ± 0.19	75.72 ± 0.24	78.09 ± 0.19	77.25 ± 0.19	71.11 ± 0.24	**79.10 ± 0.14**	77.01 ± 0.16	75.05 ± 0.23	72.41 ± 0.20	78.88 ± 0.23
BioASQ	81.66 ± 0.48	81.58 ± 0.56	84.54 ± 0.44	82.20 ± 0.39	81.90 ± 0.61	83.60 ± 0.38	79.55 ± 0.44	80.91 ± 0.57	69.64 ± 0.49	**84.24 ± 0.41**
TriviaQA	85.44 ± 0.10	84.88 ± 0.19	86.10 ± 0.10	84.61 ± 0.14	83.31 ± 0.11	86.29 ± 0.11	84.29 ± 0.11	84.07 ± 0.09	81.47 ± 0.11	**86.93 ± 0.11**
Average	79.99	79.63	81.63	80.15	78.15	81.48	78.47	78.97	73.26	**82.11**

**Table 2:** Performance (AUARC, %) comparison of various uncertainty metrics. All results are presented as percentages. For each model–dataset combination, the best performance is highlighted in bold, and the second-best performance is underlined.

Dataset	Entropy-Based Methods			Graph-Based Methods				Consistency-Based Methods		Ours
	SE	DSE	KLE	Ecc	EigV	Deg	D-UE	NSS	SEU	RSU
Llama-3.2-1B										
NQ	27.54 ± 0.63	27.43 ± 0.63	27.08 ± 0.58	27.75 ± 0.49	26.50 ± 0.53	28.10 ± 0.57	25.91 ± 0.52	27.20 ± 0.54	23.70 ± 0.50	**28.66 ± 0.56**
CoQA	86.97 ± 0.28	86.32 ± 0.16	87.65 ± 0.11	87.25 ± 0.14	85.28 ± 0.12	**87.98 ± 0.28**	87.45 ± 0.10	86.08 ± 0.26	85.80 ± 0.11	87.89 ± 0.16
BioASQ	71.25 ± 0.86	71.01 ± 0.85	70.64 ± 0.93	70.63 ± 0.90	69.37 ± 0.88	70.88 ± 0.83	70.11 ± 0.86	70.62 ± 0.91	65.72 ± 0.81	**71.33 ± 0.82**
TriviaQA	52.04 ± 0.24	51.48 ± 0.23	51.39 ± 0.37	51.55 ± 0.24	49.35 ± 0.21	52.22 ± 0.24	50.47 ± 0.21	50.89 ± 0.23	48.30 ± 0.22	**52.46 ± 0.26**
Average	59.45	59.06	59.19	59.30	57.63	59.80	58.49	58.70	55.88	**60.09**
Llama-3.1-8B										
NQ	51.74 ± 1.02	51.10 ± 1.11	51.39 ± 1.06	51.11 ± 0.90	49.51 ± 1.00	52.10 ± 0.92	49.66 ± 0.97	50.71 ± 1.20	46.83 ± 0.81	**52.27 ± 0.88**
CoQA	94.74 ± 0.29	94.79 ± 0.34	96.02 ± 0.19	95.80 ± 0.17	94.13 ± 0.24	96.30 ± 0.15	95.92 ± 0.16	94.69 ± 0.18	95.15 ± 0.15	**96.39 ± 0.15**
BioASQ	82.48 ± 0.77	82.30 ± 0.80	83.57 ± 0.67	82.97 ± 0.63	81.05 ± 0.79	83.94 ± 0.53	82.82 ± 0.54	83.21 ± 0.37	81.84 ± 0.36	**84.23 ± 0.49**
TriviaQA	84.12 ± 0.31	83.60 ± 0.33	84.18 ± 0.32	83.85 ± 0.32	82.50 ± 0.33	84.49 ± 0.29	83.57 ± 0.33	83.21 ± 0.37	81.84 ± 0.36	**84.77 ± 0.32**
Average	78.27	77.95	78.79	78.43	76.80	79.21	77.99	77.96	76.42	**79.42**
Mistral-7B-v0.3										
NQ	51.96 ± 0.61	51.49 ± 0.71	52.53 ± 0.50	52.22 ± 0.60	51.16 ± 0.56	52.75 ± 0.48	52.01 ± 0.51	51.27 ± 0.70	49.52 ± 0.64	**53.19 ± 0.58**
CoQA	92.83 ± 0.26	93.11 ± 0.22	94.29 ± 0.20	94.17 ± 0.16	91.95 ± 0.32	94.47 ± 0.13	94.32 ± 0.16	93.02 ± 0.18	93.28 ± 0.17	**94.63 ± 0.16**
BioASQ	80.46 ± 0.77	80.07 ± 0.66	**82.27 ± 0.70**	81.07 ± 0.59	80.34 ± 0.86	81.65 ± 0.63	80.18 ± 0.61	80.05 ± 0.78	73.21 ± 0.62	82.18 ± 0.61
TriviaQA	82.58 ± 0.25	82.53 ± 0.31	83.02 ± 0.25	82.31 ± 0.26	81.81 ± 0.33	83.11 ± 0.30	82.11 ± 0.30	82.23 ± 0.37	79.48 ± 0.35	**84.26 ± 0.33**
Average	76.96	76.80	78.03	77.44	76.32	78.00	77.16	76.64	73.87	**78.57**
Mistral-Nemo-12B										
NQ	51.32 ± 1.30	51.27 ± 1.17	52.12 ± 1.16	51.18 ± 1.10	50.47 ± 1.28	51.70 ± 1.12	49.83 ± 1.12	50.82 ± 1.39	47.12 ± 1.02	**52.28 ± 1.14**
CoQA	93.35 ± 0.24	93.15 ± 0.24	94.24 ± 0.18	94.15 ± 0.17	91.82 ± 0.17	94.54 ± 0.13	94.10 ± 0.14	93.02 ± 0.23	93.03 ± 0.18	**94.98 ± 0.16**
BioASQ	82.31 ± 0.56	82.00 ± 0.66	83.55 ± 0.53	82.33 ± 0.53	81.63 ± 0.70	83.50 ± 0.45	81.49 ± 0.56	81.73 ± 0.59	76.35 ± 0.54	**84.28 ± 0.60**
TriviaQA	85.35 ± 0.26	85.07 ± 0.38	85.85 ± 0.32	85.14 ± 0.26	84.14 ± 0.32	85.93 ± 0.27	85.14 ± 0.27	84.63 ± 0.27	83.31 ± 0.27	**86.34 ± 0.30**
Average	78.08	77.87	78.94	78.20	77.02	78.92	77.64	77.55	74.95	**79.47**

**Table 3:** Effect of the Rényi order α on AUROC (%) using Llama-3.2-1B. Results are reported as mean ± standard deviation over multiple runs. The best performance is highlighted in bold.

α	0.2	0.5	1.01	2	5	10
NQ	73.25 ± 0.68	74.68 ± 0.64	75.64 ± 0.52	**77.90 ± 0.54**	76.91 ± 0.54	76.32 ± 0.56
CoQA	73.11 ± 0.38	74.33 ± 0.32	74.81 ± 0.28	**75.75 ± 0.28**	75.36 ± 0.29	74.78 ± 0.30
BioASQ	86.20 ± 0.48	86.77 ± 0.49	87.23 ± 0.40	**87.55 ± 0.39**	87.36 ± 0.40	87.34 ± 0.42
TriviaQA	79.92 ± 0.26	80.18 ± 0.26	80.76 ± 0.17	**82.23 ± 0.16**	81.45 ± 0.16	81.42 ± 0.18
Average	78.12	78.99	79.61	**80.86**	80.27	79.97

**Table 4:** Effect of the fusion weight η on AUROC (%) using Llama-3.2-1B. Results are reported as mean ± standard deviation. The best performance is highlighted in bold.

η	0.01	0.25	0.5	0.75	0.99
NQ	76.98 ± 0.54	77.39 ± 0.55	**77.90 ± 0.54**	76.23 ± 0.62	75.31 ± 0.58
CoQA	75.21 ± 0.30	75.43 ± 0.28	**75.75 ± 0.28**	75.02 ± 0.33	74.71 ± 0.36
BioASQ	87.41 ± 0.42	**87.57 ± 0.41**	87.55 ± 0.39	87.23 ± 0.39	86.45 ± 0.40
TriviaQA	81.88 ± 0.16	82.16 ± 0.17	**82.23 ± 0.16**	81.38 ± 0.19	81.24 ± 0.20
Average	80.37	80.64	**80.86**	79.97	79.43

**Table 5:** Comparison between soft and hard semantic community assignments in RSU using Llama-3.2-1B. Results are reported as AUROC (%) with mean ± standard deviation. The best performance is highlighted in bold.

Dataset	Soft Community	Hard Community
NQ	**77.90 ± 0.54**	75.69 ± 0.55
CoQA	**75.75 ± 0.28**	74.31 ± 0.30
BioASQ	**87.55 ± 0.39**	86.36 ± 0.40
TriviaQA	**82.23 ± 0.16**	81.23 ± 0.17
Average	**80.86**	79.40

**Table 6:** Sensitivity of RSU to the number of sampled responses *N*. Results are reported as AUROC (%) with mean ± standard deviation using Llama-3.2-1B. The best performance is highlighted in bold.

*N*	3	5	10	20	50
NQ	69.38 ± 0.56	74.55 ± 0.56	**77.90 ± 0.54**	77.91 ± 0.54	77.92 ± 0.56
CoQA	67.51 ± 0.33	73.42 ± 0.30	**75.75 ± 0.28**	75.82 ± 0.29	75.81 ± 0.30
BioASQ	78.29 ± 0.41	83.61 ± 0.40	**87.55 ± 0.39**	87.55 ± 0.41	87.52 ± 0.43
TriviaQA	76.45 ± 0.22	78.22 ± 0.20	**82.23 ± 0.16**	82.20 ± 0.16	82.28 ± 0.17
Average	72.91	77.45	**80.86**	80.87	80.88

## Data Availability

This study evaluates semantic uncertainty estimation using publicly available question-answering benchmarks and pretrained language models. All evaluation datasets are publicly accessible from their original sources. The sampled model responses used for uncertainty estimation are generated following the experimental protocols described in the paper and can be fully reproduced using the same prompts, decoding configurations, and pretrained models. Sentence embedding models and natural language inference models employed in this work are also publicly available. The code for semantic similarity construction, soft community inference, and Rényi spectral uncertainty computation will be available from the corresponding authors upon reasonable request.

## Appendix A. Proofs of Theoretical Properties

In this appendix, we provide proofs for the theoretical properties of the Rényi spectral uncertainty measure introduced in Section 3.5. Let $\tilde{G}$ denote a unit-trace positive semi-definite matrix with eigenvalues ${\left\{\lambda_{i}\right\}}_{i=1}^{d}$, which define a probability distribution over spectral modes, and let α>0, α≠1.

**Proof** **of** **Proposition** **1** **(Degeneracy).** If $\tilde{G}$ has rank one, then its spectrum satisfies $\lambda_{1}=1$ and $\lambda_{i}=0$ for all i>1. Substituting into the definition of Rényi entropy yields

(A1) $$U_{\alpha }\left(\tilde{G}\right)=\frac{1}{1-\alpha }\log\left(\sum_{i=1}^{d}\lambda_{i}^{\alpha }\right)=\frac{1}{1-\alpha }\log\left(1\right)=0.$$

Thus, the Rényi spectral uncertainty vanishes for rank-one kernels, completing the proof. □

**Proof** **of** **Proposition** **2** **(Unitary** **Invariance).** Let *U* be an orthogonal matrix. Since $\tilde{G}$ is symmetric, the matrices $\tilde{G}$ and $U\tilde{G}U^{⊤}$ share the same eigenvalue spectrum. Because $U_{\alpha }\left(\tilde{G}\right)$ depends only on the eigenvalues, it follows immediately that

(A2) $$U_{\alpha }\left(\tilde{G}\right)=U_{\alpha }\left(U\tilde{G}U^{⊤}\right).$$

This establishes unitary invariance. □

**Proof** **of** **Proposition** **3** **(Monotonicity** **under** **Spectral** **Dispersion).** For fixed trace $\sum_{i}\lambda_{i}=1$, the Rényi entropy

(A3) $$H_{\alpha }\left(\lambda \right)=\frac{1}{1-\alpha }\log\sum_{i}\lambda_{i}^{\alpha }$$

is a Schur-concave function of the eigenvalue vector λ for all α>0. Therefore, if λ majorizes μ, then $H_{\alpha }\left(\lambda \right)\leq H_{\alpha }\left(\mu \right)$. This implies that Rényi spectral uncertainty increases as the eigenvalue distribution becomes more uniform, formalizing the notion that greater spectral dispersion corresponds to higher semantic uncertainty. □

**Proof** **of** **Proposition** **4** **(Order** **Sensitivity).** Consider two Rényi orders $\alpha_{1}<\alpha_{2}$. For a fixed eigenvalue distribution, larger values of α assign greater relative weight to larger eigenvalues in the sum $\sum_{i}\lambda_{i}^{\alpha }$. This follows from the fact that, for λ∈(0,1), the function $\lambda^{\alpha }$ decreases monotonically with increasing α, thereby amplifying the contribution of dominant spectral components. As a result, $U_{\alpha_{2}}$ becomes increasingly dominated by the largest eigenvalues, whereas $U_{\alpha_{1}}$ remains more sensitive to long-tail spectral variation, completing the proof. □
